# The Large Phenotypic Spectrum of Fabry Disease Requires Graduated Diagnosis and Personalized Therapy: A Meta-Analysis Can Help to Differentiate Missense Mutations

**DOI:** 10.3390/ijms17122010

**Published:** 2016-12-01

**Authors:** Valentina Citro, Marco Cammisa, Ludovica Liguori, Chiara Cimmaruta, Jan Lukas, Maria Vittoria Cubellis, Giuseppina Andreotti

**Affiliations:** 1 Dipartimento di Biologia, Università Federico II, 80126 Napoli, Italy; vale.ctr@gmail.com (V.C.); chiaracimmaruta@yahoo.it (C.C.); 2 Istituto di Genetica e Biofisica ‘A. Buzzati-Traverso’, CNR, 80131 Napoli, Italy; cammisamarco.py@gmail.com; 3 Istituto di Chimica Biomolecolare, CNR, 80078 Pozzuoli, Italy; lud.liguori@gmail.com (L.L.); gandreotti@icb.cnr.it (G.A.); 4 Albrecht-Kossel-Institute for Neuroregeneration, University Rostock Medical Center, 18147 Rostock, Germany

**Keywords:** Fabry disease/drug therapy, α-galactosidase, pharmacological chaperones, 1-deoxynojirimycin

## Abstract

Fabry disease is caused by mutations in the *GLA* gene and is characterized by a large genotypic and phenotypic spectrum. Missense mutations pose a special problem for graduating diagnosis and choosing a cost-effective therapy. Some mutants retain enzymatic activity, but are less stable than the wild type protein. These mutants can be stabilized by small molecules which are defined as pharmacological chaperones. The first chaperone to reach clinical trial is 1-deoxygalactonojirimycin, but others have been tested in vitro. Residual activity of *GLA* mutants has been measured in the presence or absence of pharmacological chaperones by several authors. Data obtained from transfected cells correlate with those obtained in cells derived from patients, regardless of whether 1-deoxygalactonojirimycin was present or not. The extent to which missense mutations respond to 1-deoxygalactonojirimycin is variable and a reference table of the results obtained by independent groups that is provided with this paper can facilitate the choice of eligible patients. A review of other pharmacological chaperones is provided as well. Frequent mutations can have residual activity as low as one-fourth of normal enzyme in vitro. The reference table with residual activity of the mutants facilitates the identification of non-pathological variants.

## 1. Introduction

Fabry disease (FD, OMIM #301500) is a rare pathology, but accounts for 8.8% of the patients affected by inherited disorders of metabolism [1] and is the second most common lysosomal storage disorder [2]. FD is caused by those mutations in the *GLA* gene that result in a deficiency of the protein product, lysosomal α-galactosidase (AGAL Uniprot: AGAL_HUMAN P06280; EC: 3.2.1.22), and the accumulation of its substrates. The real incidence of FD is difficult to establish. It was estimated at 1 in 100,000 [3].

Screening of various at-risk populations, patients with renal failure [4,5], stroke [6], and cardiomyopathy [7,8], have shown a significant prevalence of FD in symptomatic population. *GLA* gene variations have been found in newborn screening with a frequency as high as 1 in 1200 or 1 in 3100 [9,10]. Some of the found variations remain unclear with respect to clinical significance.

Although *GLA* is located on X chromosome (Xq22.1), heterozygous females can be symptomatic. This is due to random inactivation and lack of cross-correction that occurs in other lysosomal storage disorders such as mucopolysaccharidosis type II [11]. Random X-chromosome inactivation in heterozygous females leads to a mosaic of cells, half of which express wild-type AGAL. Under these circumstances, female patients have mild or no signs of the disease. In some cases, however, a skewed inactivation, which occurs for unknown reasons, leads to the preferential expression either of the chromosome carrying the wild type or the mutant *GLA*. Under these circumstances, female patients can be as severely affected as much as the male patients carrying the same mutation [12].

AGAL is a homodimeric glycoprotein with 429 amino acids per chain and shares structural similarities with the other lysosomal glycosidases. It catalyzes the removal of α-galactosyl residues from glycosphingolipids, in particular globotriaosylceramide, Gb3 or GL-3 (also known as ceramide trihexoside). Its products are lactosylceramide and galactose. Gb3 mainly occurs in the endothelial, kidney, heart, and nervous cells and there is evidence suggesting its involvement in the renal pathology [13,14], but the underlying mechanism remains largely unknown [15]. Gb3 and its isoforms based on ceramide modification are detectable in blood and urinary samples [16,17] from the patients for the use as diagnostic and prognostic biomarkers following and supporting genetic testing. Meanwhile, a deacylated metabolite of Gb3, globotriaosylsphingosine (lyso-Gb3) has emerged as a superior biomarker demonstrating higher sensitivity than Gb3 and a good correlation to the FD phenotype [18].

FD is characterized by a large phenotypic spectrum, with mildly and severely affected patients, and shares many symptoms with common diseases. In severe cases, often referred to as classic FD, the first specific signs appearing in childhood or adolescence are angiokeratoma, cornea verticillata, neuropathic pain, acroparesthesias and hypohidrosis. These are followed by progressive proteinuric renal insufficiency, rhythm and conductance disorders with progressive hypertrophic cardiomyopathy and cerebrovascular stroke [19,20,21]. In mild cases, often referred to as atypical FD, only some symptoms are present, usually the cardiac ones. The Mainz Severity Score Index (MSSI) [22,23] was developed to measure the severity of FD and to monitor the clinical course of the disease in response to therapy. The MSSI includes four components or sub-scores that assess the general, neurological, cardiovascular and renal signs and symptoms. Although the MSSI score is able to differentiate FD from other severe debilitating diseases, a minor, still significant overlap, in particular for cardiac sub-scores, between healthy and FD affected persons was observed. MSSI was originally developed for classic FD. Other tools, such as Fabry Disease Severity Scoring System (DS3) [24] and Fabry STabilization indEX (FASTEX) [25], were subsequently developed to cover a broader range of cases.

The broad heterogeneous symptom spectrum might be due in part to genetic modifiers and other extra-genetic (epigenetic, environmental) factors that are currently discussed [26,27].

Enzyme replacement therapy (ERT) has been approved for the last 15 years. There exist two formulations of the recombinant AGAL, agalsidase α or β, that are commercialized by Shire, Lexington, MA, USA and Genzyme, a Sanofi company, Cambridge, MA, USA respectively [28]. ERT may decrease cardiac mass [29,30,31,32] and reduce the accumulation of the substrate Gb3 in the kidney [33,34,35], but the effects on nervous system and renal function have not been definitively assessed [36,37,38,39]. Early start of ERT has been suggested because irreversible organ damage, cardiac fibrosis or severe renal dysfunction, would render the therapy ineffective [34,36,40]. Recommendations for initiation and cessation of enzyme replacement therapy in patients with Fabry disease have recently been provided by the European Fabry Working Group consensus document [28]. The effect of ERT on patients with mild mutations, which retain some residual AGAL activity, has not been considered separately [41]. This is unfortunate because ERT is not the only possible therapy for FD. A new approach with pharmacological chaperones (PC) has been proposed and a small molecular weight molecule is on the verge of being approved with the commercial name of Galafold™. This drug is an iminosugar, which closely resembles the natural product of AGAL galactose, and has been known by different names, 1-deoxygalactonojirimycin (DGJ), migalastat, AMIGAL, AT1001. DGJ inhibits reversibly AGAL at nanomolar concentrations, but stabilizes the wild type enzyme in vitro against thermal [42] and chemical induced denaturation [43] too. DGJ can be used in synergy with ERT either co-administrating both drugs intravenously or one orally (DGJ) and the other intravenously (recombinant enzyme). DGJ prolongs the half-life of AGAL in vivo, both in mouse models and in humans and leads to an improved clearance of Gb3 [44,45,46].

DGJ can be used for a stand-alone oral therapy of FD for specific missense genotypes. The efficacy of DGJ was tested in vitro, ex vivo, in cells derived from patients, and in vivo. Oral administration of DGJ reduces Gb3 in kidney, heart and skin of Fabry transgenic mice carrying the responsive human mutation R301Q [47]. When administered with an oral dose of 150 mg, it was well tolerated, increased AGAL activity [48] and decreased plasma lyso-Gb3 [47] in the majority of the patients with responsive *GLA* mutations. Interestingly, the best results are obtained when an intermittent regimen is used. The results of a clinical trial phase 3 study carried out on males and females affected by FD has been recently published. Patients received 150 mg of Galafold™ or placebo every other day. The study began with six months of double-blind administration and proceeded with 6 + 12 months of open-label administration. Although the authors conclude their abstract stating quite cautiously that “the percentage of patients who had a response at 6 months did not differ significantly between the migalastat (DGJ) group and the placebo group”, promising results are shown. A reduction of the number of Gb3 inclusions per kidney interstitial capillary as well as a reduction of plasma lyso-Gb3 were observed [49].

More than 700 variants have been reported in HGMD for the *GLA* gene so far and, differently from other lysosomal disorders such as Gaucher, there are not prevalent mutations, on the contrary most are usually found only in a single family. The number of missense mutations, 467 described so far, is a surprisingly high value for a medium size protein, such as AGAL. In order to appreciate this finding it should be considered that more than 70,000 missense mutations affecting proteins associated to human diseases have been reported, with seven variants per protein on average. The large number of missense mutations poses several problems for making a diagnosis and initiating the most appropriate therapy. Recently, it was proposed to use residual activity measured in vitro to classify mutations. We wish to contribute to the evaluation of such a proposal with the first meta-analysis of the residual activity of *GLA* missense mutations measured by several independent research groups employing different protocols, either ex vivo, in cells derived from patients, or in vitro, in transiently transfected cells. Results covering 317 of missense mutants, mostly cases reported in HGMD and associated to FD, were collected. Data were obtained in the absence or in the presence of DGJ. For this reason, our analysis provides an independent perspective on the amenability to pharmacological chaperones. In addition to this we reviewed other small molecules that were reported to have a stabilizing effect on some *GLA* missense mutations in vitro and might be developed to act in synergy or as an alternative to DGJ.

## 2. Results

### Meta-Analysis of Data Reporting Residual Activity and Responsiveness to DGJ of GLA Missense Mutations

Several independent groups have tested the effect of DGJ on AGAL mutants, administering the drug to cells derived from patients, or most frequently, to HEK293 or COS cell transiently transfected with expression plasmids. The enhancement of enzyme levels and that of the total enzyme activity is monitored in the cells extracts and is regarded as a proof of the stabilization of the mutant in the cell by DGJ. Residual activity is normalized by the total amount of protein in the cell and should not be confused with specific activity, which is normalized by the amount of AGAL. Residual activity is influenced by the stability of the mutant in the cell and by its specific activity. In general, a fixed concentration of DGJ was used, usually 20 μM, in some cases, however, IC_50_ was determined and the optimal concentration was used. The results gathered from literature are reported in Supplementary File S1 and the methods employed in each study are summarized in Table 1.

The last criteria for responsiveness were adopted for the clinical trial phase 3 published in 2016 [49] and require a relative increase in AGAL activity ≥1.2-fold above baseline and an absolute increase in AGAL ≥ 3% of wild type after incubation with 10 μM DGJ. The concentration of 10 μM is the Cmax concentration in plasma when patients are treated with 150 mg of DGJ, as was the case in clinical trials [47,49]. Ten micromolar, however, is not the highest concentration that can be safely reached in plasma [59,61] and the data obtained before 2016 with 20 μM DGJ, can still be useful to choose eligible patients.

**Figure 1:**
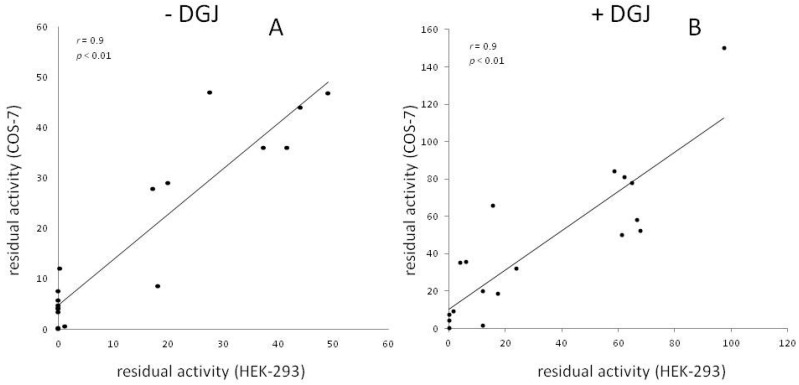
The residual activity of mutants transiently transfected and expressed in COS7 and in HEK293 is shown. In the case that multiple reports are available for a given mutant and a given recipient cell type, the average value was plotted. Results in the absence of DGJ (**A**) or in the presence of DGJ (**B**) are reported.

In vitro results are robust and do not depend on the type of recipient cells used for transfection (Figure 1).

On the other hand, residual activity measured ex vivo varies among individuals and type of cells. A few examples of the levels measured in white blood cells are provided with the average, standard deviation and number of individuals: E66Q 42.3 ± 12.5 (*n* = 9) [62]; A143T 35.9 ± 7.2 (*n* = 4), R112H 7.2 ± 7.0 (*n* = 5), R301Q 7.3 ± 2.7 (*n* = 6), R356W 1.2 ± 1.9 (*n* = 4) (Supplementary File S1).

**Figure 2:**
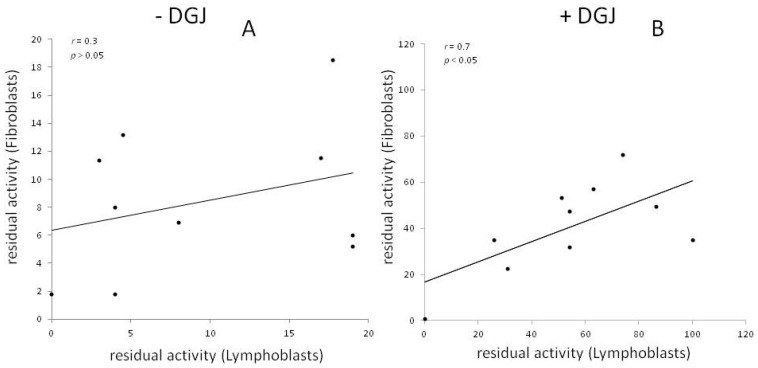
The residual activity of mutants measured in fibroblasts and lymphoblasts derived from patients is shown. In case that multiple reports are available for a given mutant and a given cell type, the average value was plotted. Results in the absence of DGJ (**A**) or in the presence of DGJ (**B**) are reported.

Figure 2 shows the average residual activity measured in lymphoblasts or in fibroblasts harboring the same mutation. A moderate yet statistically significant correlation of the data is observed only in the presence of DGJ.

**Figure 3:**
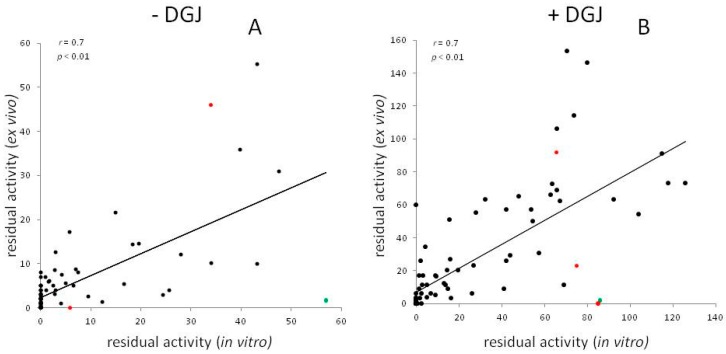
The residual activity of mutants transiently transfected and expressed in vitro and derived from patient cells is shown. In case that multiple reports are available for a given mutant, the average value was plotted. Results in the absence of DGJ (**A**) or in the presence of DGJ (**B**) are reported. Mutations affecting a site of splicing and corresponding to G183, are represented by red symbols, G128E is represented by a green symbol.

Figure 3 compares the residual activity measured ex vivo in cells derived from patients, (mostly lymphocytes or, in a few cases, fibroblasts), with that measured, in vitro, in transfected cells (HEK293, COS7 or COS1). For each mutant, the averages among results obtained by different authors without (Figure 3A) or with DGJ (Figure 3B) was determined. It can be observed that the residual activity measured in cells derived from patients tends to be lower than that measured in transfected cells, in particular in the absence of DGJ. A few examples are provided indicating in vitro result in HEK293H cells, ex vivo results in leucocytes with average, standard deviation and number of individuals: L180F: 32.4%, 6.0 ± 2.0 (*n* = 2); N215S: 39.5%, 27.1 ± 16.3 (*n* = 10); and I253T: 73.0%, 22.6 ± 6.9 (*n* = 3).

However, the residual activities in vitro and ex vivo correlate (Figure 3A *r* = 0.7, *p* < 0.01; Figure 3B *r* = 0.7, *p* < 0.01). Thus, it can be concluded that tests in vitro can generally recapitulate the residual activity ex vivo and responsiveness to DGJ.

When this manuscript had been completed, we became aware of a recent publication that reports residual activity of AGAL mutants expressed in HEK293 cells and tested with DGJ 10 μM [63]. These data correlate with those reported in Supplementary File S1 (−DGJ *r* = 0.8, *p* < 0.000001; +DGJ *r* = 0.7, *p* < 0.000001).

Test in vitro have a limitation because they cannot account for the effect of exonic mutation on splicing. In fact, mutants are encoded by plasmids that do not contain introns. It is interesting to analyze the case of the mutations affecting a site of splicing and corresponding to G183 represented by red symbols in Figure 3. Substitution of GLY by SER results in a mutant that does not retain activity in cells derived from patients and does not recover activity with DGJ. The same mutant recovers activity with DGJ in vitro. We can hypothesize that the drug has a stabilizing effect on the protein, but cannot correct the effect on splicing. On the other hand, G183A and G183D are responsive to the drug both in vitro and ex vivo suggesting that these mutations mainly affect the protein, but not the splicing. K213M might be another example where splicing could play a role to explain the in vitro and ex vivo differences. Mutations not occurring at splicing sites can have effect on the maturation of RNA too. We suspect that this might be the case for G128E, green symbol in Figure 3, because very low residual activity was measured by several authors in cells derived from patients, either in the presence or in the absence of DGJ, whereas the mutation retains residual activity and is responsive to DGJ in vitro. Although a putative consensus for an exonic splicing enhancer including the triplet 128 was found, further experiments are needed to confirm the influence of the mutation on RNA processing.

We report the score obtained by a position specific substitution matrix (PSSM) that measures whether the mutation was tolerated during the evolution of homologous proteins in Supplementary File S2. Mutations affecting the active site, as expected, have no residual activity and do not respond to DGJ, mutations occurring at non-conserved sites tend to be responsive.

Predictions were obtained with Web based Polyphen2 using HumDiv or HumVar as the training set and are reported in Supplementary File S2. Both sets use disease mutations in UniprotKB as positive controls, but differ for the negative control set. HumDiv uses differences between human proteins and their closely related mammalian homologs, whereas HumVar uses common human SNPs (MAF > 1%) without annotated involvement in disease. HumVar-trained model is suitable to distinguish mild mutations, the HumDiv-trained model, considers also mild mutations as deleterious one. Although, on average, the residual activity of mutations that are predicted as probably damaging with both training sets is very different from the residual activity of mutations predicted as benign, exceptions can be observed in particular with HumVar-trained model (Table 2).

Other in silico approaches based on the structural features of AGAL, some from our group [64], have been attempted [65,66], to predict the severity of FD genotypes. We believe that data obtained in vitro should always be preferred whenever available.

In Supplementary File S2, all missense variants of *GLA* described in ExAC [67] are reported. ExAC summarizes exome sequencing data from a wide variety of large-scale sequencing projects. Variants reported in this database, in particular those observed with higher frequency, are likely to be non-pathogenic. The mean residual activity measured in vitro for a subset of ExAC variants, i.e., those observed in more than one male is reported in Table 2. The number of hemizygous individuals reported in ExAC, the PSSM score, Polyphen2 prediction and the reference found in HGMD are reported in the same table.

R118C is the variant with the lowest residual activity, only 24.5% of wild type when tested in transiently transfected cells. It is relatively frequent in the European population, but it is predicted as deleterious both by Polyphen_Humvar and Polyphen_Humdiv. Oliveira and colleagues reviewed the clinical, biochemical and histopathology data obtained from 22 individual carriers and reached the conclusion that it “does not segregate with FD manifestations at least in a highly-penetrant Mendelian fashion”, but might be a risk factor for stroke [73]. In accordance with this, low levels of lyso-Gb3, a biomarker of FD, were measured in the carriers [2]. R118C is considered amenable to DGJ according to galafold amenability table [63]. R118C was tested with DGJ and with Rosiglitazone by Lukas et al. [74]. Although in terms of activity fold increase, the effect of mono-therapy with either drug was small, the combinatorial effect was significantly higher.

A143T has an average residual activity of approximately 39.7% of wild type. Brand and coworkers [75] analyzed 15 females and 10 males carrying this mutation. They observed that female and male A143T carriers showed less organ involvement in comparison to FD patients with other missense mutations and those suffering from stroke/TIA showed no further FD-typical organ manifestations. They came to the conclusion that “A143T seems not to be causal for FD, but rather a genetic variant of unknown significance or a genetic modifier”. A143T is considered amenable for the therapy with DGJ according to the galafold amenability table.

E66Q has specific activity, *V*max, and affinity for the artificial substrate 4-methylumbelliferyl-α-galactopyranoside, *K*m, similar to those of wild type, but residual activity in transfected cells is approximately one half of the wild type, possibly because the stability at neutral pH is reduced [52]. The mutation is relatively frequent in East Asian population. Sakuraba and coworkers measured the activity in 20 Japanese or Korean male carriers with renal and cardiovascular disorders and found 13% to 26% of the normal mean values for plasma and 24% to 65% of the normal mean values for white blood cells, but the lyso-Gb3 levels were as low as those of healthy controls and no inclusion bodies were found [62]. Hu and co-workers found that the mutation segregated with renal disease in a very large Chinese family, but they did not measure the accumulation of the substrate or of lyso-Gb3 in the same patients [76]. The involvement in cardiovascular disease has also been suspected, but no accumulation of Gb3 was found in the heart of a patient carrying E66Q [77]. The association between E66Q and the risk cerebral small-vessel occlusion is debated [78,79]. In conclusion, pathogenicity of E66Q is still vexata quaestio. E66Q is considered non amenable for DGJ according to galafold amenability table, but an increase in activity upon drug administration was measured by other authors [52,55,57,80].

D313Y was first associated to classic phenotype [71]. Subsequent data clinically and biochemically indicated that D313Y should be considered a variant [81]. Cardiac, nephrological, neurological, laboratory and quality of life data were collected from carriers of D313Y with a 4-year follow up and the results indicated that the mutation is non pathological. Very low levels of lyso-Gb3 were found [2]. The opinion that D313Y is a non-pathological variant is supported by the fact that its frequency of 0.4% in the non-Finnish European population is much higher than the prevalence of FD in the same population. D313Y is considered amenable for DGJ.

For other mutations such as S126G and N139S, the clinical picture did not include specific signs of FD [69,70]. Both mutations are considered amenable for DGJ.

This survey would suggest that a residual activity higher than 25% can indicate a non-pathological variant. Nonetheless, when considering administration of a therapy, if any, clinicians should be aware of the fact that the severity of the disease depends not only on the damage caused to the protein itself by the mutation, but also by other factors, which regrettably have not yet been clarified. It should be considered that the phenotype can differ even among the members of the same family [82] and that the residual activity in plasma or in white blood cells can vary largely in people carrying the same mutation [62].

N215S is a mutation affecting glycosylation of the AGAL enzyme [83]. It presents with a proportionally high residual activity >25% of normal. It is considered a distinct sub-type of FD due to its elevated prevalence compared to non-N215S FD cases and late-onset occurrence [60,84]. Interestingly, this variant has never been scrutinized to cause a pathogenic phenotype. By contrast, it is believed to cause a specific cardiac phenotype. Other cardiac-prone mutations might exist, e.g., the so-called IVS4 + 919G > A splice mutation highly prevalent in the Taiwanese population. A clinical trial investigating the long term clinical course of N215S patients is currently ongoing (clinicaltrials.gov, NCT01429597). In this case, the diagnostic and prognostic value of biomarker lyso-Gb3 can be appreciated. While apparently all (genetically) found patients were identified it was demonstrated that Gb3 was normal in a great fraction of patients. Meehan et al. [85] showed that N215S was present in a patient with renal manifestation and is, thus, suggestive to cause mainly cardiac and renal symptoms. N215S is amenable for DGJ.

## 3. Future Perspectives for Therapy

DGJ is a promising drug, but it might not be the ideal drug yet. DGJ inhibits AGAL at nanomolar concentration and stabilizes it at micromolar concentrations. Therefore a continuous exposure to the drug can promote AGAL levels, but not AGAL intracellular activity. Reduction of Gb3 concentration was not observed in fibroblasts derived from patients carrying the mutations R301Q or L300P and incubated with DGJ for 10 days, but was observed if the incubation of seven days with the drug was followed by a three day wash-out [55].

The discovery of DGJ was the result of an educated guess, and not of a methodical screening [86]. In fact, DGJ is a glycomimetic with a six-atom ring and very closely resembles the galactose, which is the natural product and inhibitor, and also the first chaperone described for AGAL [87]. A more systematic search was started with the aim of finding other drugs that might have a better ratio between the stabilizing and the inhibitory effect. Most of the molecules considered so far are glycomimetics as DGJ itself. DGJ is an amine and is positively charged at neutral pH. In order to facilitate its diffusion through membranes, alkylation was proposed [88]. Contrary to what was observed for analogous iminosugars active on other lysosomal glycosidases, alkyl-DGJ derivatives had a lower affinity for AGAL and apparently a lower chaperoning potential probably because one important hydrogen-bond, the one established between the heterocyclic NH proton and D170 of AGAL, is lost.

On the contrary, aryl DGJ-derivatives (1-deoxygalactonojirimycin-arylthioureas) that form a hydrogen bond between the aryl-N’H thiourea proton and D231 of AGAL, act as reversible inhibitors and chaperones. When tested at 30 μM concentration on Q279E or R301Q mutants, the best candidate, namely *N*’-*p*-methoxyphenyl-DGJ-Aryl thiourea, had a seven fold higher chaperoning activity than DGJ at its optimal concentration [89].

Iminosugars characterized by a smaller, five-atom ring system, have been described [90,91]. 2,5-dideoxy-2,5-imino-d-altritol (DIA) inhibited AGAL and stabilized it against thermal denaturation and acted as a chaperone when tested on Fabry R301Q lymphoblasts although at a concentration 20 times higher than the optimal one for DGJ. The effect on Gb3 accumulation was not tested. One derivative of DIA possessing an aminomethyl group showed a chaperoning effect higher than DGJ when administered to N215S patient lymphocyte cell line at high concentration (100 μM) [92].

DGJ binds and inhibits AGAL both at neutral pH, which is required, and at acidic pH, which is not required [42]. It would be useful to find molecules that bind and stabilize AGAL mutants when they are in the neutral environment of the endoplasmic reticulum, but dissociate when the protein reaches the lysosome. This point was specifically addressed incorporating an orthoester segment into DGJ [93].

Glycomimetics require a precise dosing, whereas non carbohydrate mimetics might offer a larger therapeutic window and an improved therapeutic index. In order to look for chemically diverse drugs, a library of 230,000 diverse compounds was screened but no inhibitors or activators of a-Gal A with an IC_50_ below 50 μM were identified. Unfortunately the screening procedure relied only on an enzymatic assay carried out at pH 5.9, but not at neutral pH or on assays based on AGAL stabilization [94].

So far reversible inhibitors of AGAL that act as PC have been described. The association between the two effects, inhibition and stabilization, is avoidable because active sites are not the only targets for chaperones. Allosteric ligands might act as pharmacological chaperones, and might be more effective than reversible inhibitors, since they would perform their stabilizing action without competing with the natural substrate. Looking for allosteric PC is difficult because they do not resemble chemically known substrates. Large libraries of structurally diverse compounds should be tested and preliminary screening in silico might be functional. Allosteric ligands do not bind the active site, but one of the many pockets occurring on the surface of a protein. Therefore it is difficult to restrict the area where binding is allowed as required by structure based virtual screening. A recent screening, carried out on 10,000 molecules, showed that it is possible to find molecules that, at least in silico, preferentially bind an allosteric site than the active site. The two sites are located at the opposite sides of the catalytic domain of AGAL [95].

The PC that have been described previously are specific ligands of AGAL. They are effective on missense mutations that cause destabilization of the enzyme and, ultimately, its early degradation. Other small molecules that do not physically interact with AGAL, but have effect on proteostasis, can be considered for the treatment of these cases as well. Proteostasis regulators can be used in synergy with specific PC potentiating their action or allowing lower dosages.

The before mentioned Rosiglitazone, a Peroxisome proliferator-activated receptor gamma (PPARγ) agonist rearranges global cellular ubiquitination by inhibiting the ubiquitin-proteasome system (UPS). It displayed the highest beneficial effect on mutations with a significant residual activity (e.g., R118C and T385A) and was even more effective in combination with a PC [60]. Mechanistic studies are required to explain why other ubiquitination inhibitors such as Pyr-41 failed to increase AGAL activity. This finding might be ascribed to intense adverse effects of cellular ubiquitination inhibition caused by Pyr-41 and associated toxicological aspects.

Ambroxol, a mucolytic agent used in the treatment of respiratory diseases, was identified as an enhancer of AGAL activity. The compound has formerly been demonstrated to act as a PC on mutant Glucocerebrosidase in Gaucher disease. Even though its mechanism of action is not known it was demonstrated to increase cellular AGAL level and activity of most (DGJ−) amenable mutations (E59K, A73V, A143T, A156V, I232T, R301G, R301Q, R356W and R363H) indicating an impact on AGAL proteostasis. Ambroxol was, however, not effective as a monotherapy, but only in the combination with a PC, galactose or DGJ [60].

The synergistic effect of *N*′-*p*-methoxyphenyl-DGJ-Aryl thiourea with two proteostasis regulators, 4-phenylbutyric acid and celastrol has been assessed. The latter compound was not effective, but 4-phenylbutyric acid at 0.1 mM concentration was able to enhance the chaperoning activity of the aryl-thiourea (20 μM) on the fibroblasts harbouring Q279E [89].

The effects of lactacystin 2 μM (a proteasome inhibitor) and kifunensine 0.2 mM (an inhibitor of ER *α*-mannosidase I) on the processing on some mutants assessing the amount of protein was tested by Fan and coworkers [52] They found that some mutants responded to both drugs (F113L, N215S, and M296I), one responded only to lactacystin (E66Q), others only to kifunesine (M72V, I91T, A97V, R112H, L166V, and Q279E), and others had low or no response to either (A20P, A156V, M296V, R356W, G373D, G373S, E59K, and P146S). All mutants that are responsive to kifunensine or lactacystin are also responsive to DGJ, while A156V, M269V, R356W, and E59K are responsive only to DGJ. These results suggest that a different cocktail of drugs might be ideal for specific AGAL mutations.

## 4. Methods

Residual activities in Supplementary File S1 were obtained from the literature. In those cases where the authors did not report the normalized percentage values, the activity of the mutant in the presence of DGJ was divided by the activity of wild type AGAL multiplied by 100 (+DGJ/wild × 100). The reference wild type activity, measured in the absence of DGJ was obtained for each mutation from the appropriate paper. IC_50_ values are reported when available.

Pearson correlation coefficients and two tailed *p*-values were calculated as described by Lowry [96].

PSSM values were calculated as described [97,98]. Active site residues were identified with DrosteP [99]. Predictions were obtained with Web based Polyphen2 using HumDiv or HumVar as the training set under default conditions [100].

## Figures and Tables

**Table 1:** Experimental conditions under which DGJ responsiveness has been assessed.

Reference	Cell Type	Concentration and Incubation Time
Ishii_2000 [50]	Transfection COS1	20 μM DGJ 1 day
Spada_2006 [10]	Transfection COS7	20 μM DGJ 72 h
Shin_2007 [51]	T-cells and fibroblasts	20 μM DGJ 3 or 4 days
Ishii_2007 [52]	Lymphoblasts and fibroblasts	20 μM DGJ 5 days
Shin_2008 [53]	T-cells	20 μM DGJ 3 days
Park_2009 [54]	Transfection COS7	20 μM DGJ 2 days
Benjamin_2009 [55]	Lymphoblasts and fibroblasts	Depending on EC50 5 days
Filoni_2010 [56]	Transfection COS1 and lymphocytes	20 μM DGJ 72 h
Wu_2011 [57]	Transfection HEK293	Depending on EC50 4 to 5 days
Andreotti_2011 [58]	Transfection COS7	20 μM DGJ 48 h
Lukas_2013 [2]	Transfection HEK293H	20 μM DGJ 60 h
Giugliani_2013 [59]	Transfection HEK293	10 μM DGJ
Lukas_2016 [60]	Transfection HEK293	20 μM DGJ 60 h

**Table 2:** Putative non-pathological AGAL mutants: features and residual activity in vitro.

Mutation	No. Hemiz	−DGJ	+DGJ	PSSM	Humdiv	Humvar	Reference
L3P	4	117.7	129.4	−3	Probably damaging	Probably damaging	[60]
E66Q	3	47.6	53.66	−2	Probably damaging	Probably damaging	[68]
R118C	8	24.5	27.8	−2	Probably damaging	Possibly damaging	[10]
N139S	7	147.8	176.4	−1	Benign	Benign	[69]
S126G	18	51.3	67.4	−2	Benign	Benign	[70]
A143T	19	39.7	63.7	−1	Probably damaging	Possibly damaging	[71]
I289V	3	79.9	95	0	Probably damaging	Possibly damaging	
D313Y	129	75.5	100.3	−1	Probably damaging	Possibly damaging	[71]
R363H	3	28	65.7	−1	Benign	Benign	[72]
A368T	3	103.7	93.3	0	Benign	Benign	[2]
T385A	36	45	48.9	−2	Possibly damaging	Benign	[2]
W399S	5	53	51.5	−4	Possibly damaging	Benign	[60]

## Supplementary Materials

Supplementary materials can be found at www.mdpi.com/1422-0067/17/12/2010/s1.
